# Identification of candidate genes for *Nilaparvata lugens* (stål) resistance through genomic dissection from diverse Indigenous rice genotypes

**DOI:** 10.1186/s40529-025-00461-3

**Published:** 2025-07-14

**Authors:** Guru-Pirasanna-Pandi Govindharaj, Soumya Bharati Babu, C. Anilkumar, Debajyoti Roy, C. Parameswaran, G. Basana-Gowda, Ruchi Bansal, SD Mohapatra

**Affiliations:** 1 Crop Protection Division, ICAR-National Rice Research Institute, Cuttack, 753006 India; 2 Sri Sri University, Cuttack, 754006 India; 3 ICAR-National Bureau of Plant Genetic Resources , New Delhi, 110012 India; 4 Department of Agronomy and Plant Genetics, University of Minnesota, Minnesota, MN USA

**Keywords:** Brown planthopper, Hemiptera, Defence, SSR markers, Trait association

## Abstract

**Background:**

Rice production faces relentless threat from the brown plant hopper (*Nilaparvata lugens*), particularly in Asian subcontinents like India, where the highly damaging biotype-4 is prevalent. Developing rice varieties with long-lasting resistance is crucial to combat this threat sustainably. This study aimed to identify stable novel resistant sources and associate molecular markers with resistant loci present in the new sources of resistance. In this study, 152 rice genotypes were screened against *N. lugens*, and further genotyping was done using 82 SSR (Simple Sequence Repeat) markers linked to 28 *N. lugens*-resistant genes and QTLs.

**Results:**

After continuous screening for three seasons, 33 resistant rice genotypes (score 1) were identified. Unlike susceptible genotypes, resistant genotypes exhibited lower plant damage, nymphal survival, and honeydew excretion, with ranges of 4.60–8.90%, 11.50–24.00%, and 3.43 to 7.43 cm², respectively. However, resistant genotypes showed more feeding marks, ranging from 22.67 to 32.00 plant^− 1^. Genetic analysis indicated an average genetic diversity of 0.150 and a polymorphic information content of 0.128 for the markers. Cluster and population structure analyses classified the genotypes into three primary genetic groups. This grouping was corroborated by Principal Coordinate Analysis (PCoA), which separated susceptible, moderately resistant, and resistant genotypes into distinct components. Additionally, 87% of the genetic variation was between individuals of the populations and 13% between populations.

**Conclusion:**

Marker-trait association analysis using generalized linear models (GLM) and mixed-linear models (MLM) identified two markers viz. RM1313 (*Bph9*) and RM7 (*Qbph3*) were significantly associated with phenotypic parameters related to *N. lugens* resistance. Among these RM7 (*Qbph3*) was identified by both GLM and MLM analyses. These findings highlight the potential of identified resistant genes in rice landraces for developing durable resistant varieties against *N. lugens*.

**Supplementary Information:**

The online version contains supplementary material available at 10.1186/s40529-025-00461-3.

## Introduction

Rice (*Oryza sativa* L.) is the main dietary staple for over half of the global population, significantly contributing to their daily nutritional and caloric needs. In India, rice production is about 135.8 million tonnes, making up 40% of the country’s total food grain production. Nevertheless, India’s average rice yield of 2600 kg ha^− 1^ lags behind that of countries such as China, Indonesia, and Vietnam (Pathak et al. 2020). Hence, boosting food production is crucial to meet the increasing demand and ensure food security. However, rice cultivation faces significant challenges from various biotic and abiotic factors. In particular, insect herbivores substantially threaten rice production, with around 100 species targeting the crop, of which 20 are considered major threats. Among these, the brown planthopper, *Nilaparvata lugens* (Stål.) stands out as a devastating pest. Feeding solely on rice causes severe economic losses (70–100%) annually in Asian countries (Pandi et al. 2018; Jena et al. 2018; Horgan et al. 2020).

Both adult and nymph stages of *N. lugens* extract the plant’s phloem sap with their needle-like mouthparts, causing plant wilting and the characteristic ‘hopper-burn’ symptom. *N. lugens* also serves as a vector for viral diseases such as ragged and grassy stunt in rice (Huang et al. 2015). Presently, four biotypes of *N. lugens* are recognized, with biotype-4 posing the greatest threat to rice in the Asian subcontinent, also known as the South Asian biotype (Jena et al. 2018; Jeevanandham et al. 2023). The primary management strategies against this pest include cultivating resistant rice varieties and the application of chemical insecticides (Du et al. 2020; Pandi et al. 2021). Nevertheless, the excessive and indiscriminate use of chemical insecticides has led to *N. lugens* developing resistance or tolerance to these chemicals (Wu et al. 2018; Horgan et al. 2020).

An alternative, more sustainable approach involves utilizing host plant genetic resistance as a cost-effective and environmentally friendly method for managing *N. lugens* (Hu et al. 2016; Kumar et al. 2020). So far, 46 genes that resist *N. lugens* have been discovered and identified in rice and wild relatives; however, only a handful have been functionally characterized and incorporated into breeding programs (Kaur et al. 2024). Unfortunately, *N. lugens* biotype 4 has already surpassed the resistance found in several renowned resistant rice genotypes (Han et al. 2018; Anant et al. 2021). Since *N. lugens* feeds exclusively on rice, it can rapidly mutate to overcome resistance genes, leading to new virulent strains (Horgan et al. 2016).

Therefore, due to the ongoing evolution of the pest alongside its host plants, it is essential to seek new and effective sources of resistance from diverse resources, such as landraces and wild relatives. India is home to a rich diversity of rice germplasm and landraces, characterized by various morphological and genetic variations resistant to various stresses. Despite their lower yield, traditional landraces possess unique resistant traits, making them valuable for trait improvement (Umakanth et al. 2017; Ngangkham et al. 2018; Han et al. 2018; Kumar et al. 2020). This study aims to characterize sources or genes of resistance to *N. lugens* from Indigenous rice genotypes through phenotypic evaluation and genetic analysis, aiding in the creation of durable, resistant rice varieties that maintain resistance to *N. lugens*. We hypothesized that the diverse rice genotypes and landraces used in the present study have valuable novel resistance genes against highly virulent and dynamic biotype-4 of *N lugens*. To support the hypothesis, diverse rice genotypes and landraces were evaluated and assessed with molecular markers to identify stable resistant sources. The identified resistant source has significant scope in utilization in resistant cultivar breeding programs.

## Materials and methods

### Rice genotypes and insect culture

The seed used in our experiment was sourced from the rice gene banks at ICAR-National Rice Research Institute in Cuttack, Odisha, which represent diverse Indian rice cultivation zones. Initially, we conducted screenings of landraces for resistance to *N. lugens*, examining 152 rice genotypes, including two resistant (PTB33 and Salkathi) and one susceptible (TN1) control. The screening and phenotypic evaluations were conducted over three rainy seasons from 2021 to 2023. The initial *N. lugens* populations were gathered from the ICAR-NRRI research farm and were bred in a glasshouse under controlled conditions (28 ± 2 °C, 70 ± 5% humidity, 14:10 h light: dark cycle). The glasshouse featured an air cooling and lighting system to ensure ideal conditions. *N. lugens* were reared on the susceptible variety Taichung Native 1 (TN1) by releasing 20 pairs of adults onto 40-day-old plants and allowed to lay eggs for 24 h. The plants were then moved to rearing cages to use the emerging insects for the phenotypic and genotypic screenings.

### Phenotypic evaluation

#### Screening and scoring of rice genotypes against *N. lugens*

The Standard Seed Box Screening Test (SSST), modified from Jena et al. (2006), was utilized to assess the resistance of selected rice germplasm against *N. lugens*. The SSST method allows for the ranking and scoring (0–9) of rice germplasm based on damage inflicted by *N. lugens* nymphs (Supplementary Table 1). Every germplasm together with two resistant checks (Salkathi, PTB33) and one susceptible check (TN1), was represented by 20–25 pre-germinated seeds sown in rows within a seedbox (45 cm x 35 cm x 15 cm). Each entry had two rows in a randomized design, with three replicates per seed box. Twelve days post-germination, ten 2nd instar *N. lugens* (biotype 4) nymphs infested each seedling. Scoring on a 0–9 scale was conducted once all TN1 seedlings wilted from *N. lugens* feeding.

#### Investigation of nymph survival rate, number of feeding marks, and honeydew excretion

The viability of *N. lugens* nymphs was assessed on 30-day-old potted seedlings of each germplasm, adhering to the methodology outlined in the standard protocol (Heinrish et al. 1985), with five replicates each infested with 20 s-instar nymphs enclosed in a mylar film cage (15 cm x 30 cm). Fifteen days later, the count of surviving nymphs determined the survival rate. Assessment of *N. lugens* feeding damage was conducted following the approach outlined by Natio (1964), enhanced by Anant et al. (2021). In this method, 24 h post-nymphal feeding, rice stems underwent staining with 0.15% Rhodamine dye for 15 min. Subsequently, a trinocular stereo-zoom microscope (Nikon, SMZ745T) was utilized to quantify the stained-style probes on each genotype. in the honeydew excretion measurement, five pre-starved, newly emerged female *N. lugens* were placed on filter paper beneath each germplasm for 24 h with three replicates (Sogawa and Pathak 1970). The area of blue-rimmed spots from honeydew excretion on the filter paper was then graphically measured in cm².

### DNA extraction and markers analysis

Plant genomic DNA from young leaf tissues was extracted using the Cetyltrimethyl Ammonium Bromide (CTAB) method, following the procedure outlined by Umakanth et al. (2017) with slight modifications. The DNA quality and quantity were confirmed through 0.8% agarose gel electrophoresis and a NanoDrop OneC spectrophotometer (Thermo Fisher, USA), respectively. Then, DNA samples were diluted to a working concentration of 20 ng/µl with nuclease-free water. Subsequently, the genotype panel underwent screening for 28 *N. lugens* resistance genes and QTLs using 82 gene-linked markers. (Supplementary Table 2). PCR amplification followed the procedure outlined by Anant et al. (2021a). The PCR products were examined through 2% agarose gel electrophoresis, stained with ethidium bromide, and run alongside a 100 bp DNA ladder. Visualization was performed with a gel documentation unit (BIO-RAD, USA), and markers scored 1 or 0 based on the bands’ visibility.

### Statistical analysis

TASSEL 5.0 software was used to analyse the marker-trait association of 82 molecular markers associated with 26 *N. lugens* resistant genes using MLM (mixed linear model), GLM (general linear model), and GAPIT 3.0 for Farm CPU (a multi-locus model) (Bradbury et al. 2007). The POWER MARKER Version 3.25 software was employed to calculate the population parameters such as heterozygosity, allele count per locus, genetic diversity, frequency of the predominant allele, and the polymorphism information content of the selected markers (Liu and Muse 2005). An unweighted neighbor-joining (NJ) unrooted tree, built from 82 molecular markers linked to 26 *N. lugens* resistance genes, was created with DARwin version 6.0.21 software, which facilitated the analysis of genetic dissimilarity and distance (https://darwin.cirad.fr/). The dissimilarity index was calculated using the NEI coefficient (Nei 1973) and supported by a bootstrap value of 1000. The STRUCTURE software version 2.3.4 was utilized to analyse population structure and detect admixture within and among subpopulations in the panel. The optimal K-value was identified through the ΔK method using the Structure Harvester tool Ver.0.6.193 (Earl and VonHoldt 2012; Evanno et al. 2005). GenAlEx 6.502 (Peakall and Smouse 2006, 2012) was used to conduct principal coordinate analysis (PCoA), calculate the fixation index (Fst), and assess molecular variance (AMOVA) across and within populations. Additional statistical analyses were carried out using SAS software version 9.1.

## Results

### Phenotypic variation

A screening of 152 rice genotypes was conducted against *N. lugens*, which comprised 33 resistant (rated score 1), 70 moderately resistant (score 3), 12 moderately susceptible (score 5), 7 susceptible (score 7), and 30 highly susceptible (score 9) genotypes (Table 1). Examination of the phenotypic reactions of these rice landraces to *N. lugens* infestation showed notable differences within the panel (Table 1). Moreover, the distribution pattern of *N. lugens* resistance genes in the panel was assessed using skewness (third-degree) and kurtosis (fourth-degree) statistics, where the panel’s mean SES score was 4.37, with a standard deviation of 2.54. The population showed positive skewness, and the kurtosis value was below three, indicating a platykurtic distribution (Table 2). In the case of resistant genotypes, the plant damage (PD) percentages ranged between 4.60% and 8.90%, whereas the highly susceptible genotypes showed PD percentages from 84.83 to 100%. Nymph survival (NS) percentages in resistant genotypes varied between 11.50% and 24.00%, starkly contrasting the 74.00–96.60% NS rates observed in highly susceptible genotypes. The number of feeding marks (FM) left by *N. lugens* was highest in resistant genotypes, with 32.00 FM per plant, compared to less FM (6.30–9.77) per plant in the highly susceptible genotypes. Furthermore, the secretion of honeydew by *N. lugens* was significantly higher on the highly susceptible genotypes, measuring 43.90–58.53 cm^2^, as opposed to a mere 3.43–7.43 cm^2^ on resistant genotypes. The present study identified 31 highly resistant accessions, and based on the origin of resistance, most of these materials were from eastern India (Odisha, Jharkhand, Chhattisgarh, and Bihar). Particularly, 9 from Chhattisgarh, 5 from Odisha, 4 from Bihar, and 2 apiece from Assam, Kerala, and Uttar Pradesh.

**Table 1:** The phenotypic reaction of *N. lugens* in the rice genotypes

Sl.no.	Designation	Score	Plant damage (%)	Nymphal survival (%)	Feeding marks (Number)	Honeydew excretion (cm^2^)	Districts (States)
1	IC283013	1	12.23 (14.90)	12.23 (20.40)	26.33 (5.18)	5.13 (2.37)	Cuttack (Odisha)
2	IC283138	1	15.00 (17.15)	15.00 (22.76)	26.00 (5.15)	6.37 (2.62)	Kendrapara (Odisha)
3	IC283247	1	14.00 (13.26)	14.00 (21.96)	25.00 (5.05)	5.63 (2.48)	Puri (Odisha)
4	IC311851	1	14.83 (16.15)	14.83 (22.65)	26.03 (5.15)	5.47 (2.44)	Palghat (Kerala)
5	IC256545	1	20.17 (17.35)	20.17 (26.67)	23.10 (4.86)	3.43 (1.98)	Debagarh (Odisha)
6	IC324133	1	24.00 (16.45)	24.00 (29.30)	22.67 (4.81)	5.90 (2.53)	Darrang (Assam)
7	IC326270	1	14.97 (12.38)	14.97 (22.73)	24.60 (5.01)	5.90 (2.53)	Saiha (Mizoram)
8	IC334164	1	11.50 (16.10)	11.50 (19.80)	25.40 (5.09)	5.47 (2.44)	Ranchi (Jharkhand)
9	IC343383	1	22.00 (13.41)	22.00 (27.94)	23.77 (4.93)	4.63 (2.27)	Wayanad (Kerala)
10	IC343507	1	21.33 (17.08)	21.33 (27.46)	23.53 (4.90)	7.43 (2.82)	Mau (Uttar Pradesh)
11	IC346221	1	12.00 (12.73)	12.00 (20.18)	30.77 (5.59)	3.57 (2.02)	Madhubani (Bihar)
12	IC346232	1	11.83 (13.65)	11.83 (20.11)	32.00 (5.70)	3.63 (2.03)	Madhubani (Bihar)
13	IC346233	1	15.20 (17.14)	15.20 (22.81)	24.77 (5.03)	5.13 (2.35)	Madhubani (Bihar)
14	IC346262	1	14.50 (15.50)	14.50 (22.16)	22.83 (4.83)	5.30 (2.41)	Darbhanga (Bihar)
15	IC346890	1	16.53 (13.69)	16.53 (23.91)	25.47 (5.09)	5.13 (2.37)	Bastar (Chattisgarh)
16	IC346892	1	17.33 (16.84)	17.33 (24.33)	24.30 (4.98)	5.57 (2.45)	Bastar (Chattisgarh)
17	IC377588	1	19.17 (14.37)	19.17 (25.62)	22.93 (4.84)	4.77 (2.28)	Raipur (Chattisgarh)
18	IC426126	1	18.83 (15.96)	18.83 (25.50)	26.80 (5.21)	5.73 (2.47)	Bastar (Chattisgarh)
19	IC426128	1	14.50 (15.86)	14.50 (22.25)	28.83 (5.41)	4.37 (2.20)	Bastar (Chattisgarh)
20	IC426139	1	18.17 (13.63)	18.17 (25.08)	24.10 (4.96)	5.43 (2.41)	Dantewada (Chattisgarh)
21	IC426144	1	19.87 (17.17)	19.87 (26.36)	26.50 (5.19)	4.63 (2.26)	Bastar (Chattisgarh)
22	IC426157	1	15.10 (14.78)	15.10 (22.72)	28.27 (5.36)	4.67 (2.24)	Bastar (Chattisgarh)
23	IC438611	1	17.87 (14.01)	17.87 (24.77)	26.43 (5.18)	4.00 (2.11)	Bastar (Chattisgarh)
24	IC444008	1	16.33 (15.38)	16.33 (23.65)	25.00 (5.02)	4.97 (2.33)	Kerala
25	IC515159	1	15.33 (14.41)	15.33 (22.94)	24.93 (5.04)	4.83 (2.29)	Bargarh (Odisha)
26	IC515880	1	14.33 (16.21)	14.33 (22.10)	25.33 (5.08)	5.20 (2.39)	Tirunelveli (Tamil Nadu)
27	IC519101	1	17.33 (14.71)	17.33 (24.33)	25.77 (5.12)	6.63 (2.66)	Deoria (Uttar Pradesh)
28	IC574971	1	20.53 (15.79)	20.53 (26.84)	25.67 (5.11)	4.17 (2.15)	Lakhimpur (Assam)
29	IC752742	1	16.00 (14.71)	16.00 (23.17)	24.00 (4.95)	3.37 (1.97)	Unknown
30	IC75881	1	16.17 (17.05)	16.17 (23.31)	24.43 (4.99)	5.17 (2.38)	IIRR (Hyderabad)
31	IC75883	1	18.83 (13.17)	18.83 (25.50)	23.17 (4.86)	5.37 (2.39)	IIRR (Hyderabad)
32	PTB33	1	16.50 (15.15)	16.50 (23.77)	25.93 (5.13)	5.33 (2.41)	Kerala
33	Salkathi	1	15.53 (15.43)	15.53 (23.15)	26.67 (5.20)	6.00 (2.54)	Sambalpur (Odisha)
34	IC256523	3	25.50 (20.26)	25.50 (30.33)	16.37 (4.11)	6.83 (2.71)	Debargarh (Odisha)
35	IC256538	3	24.20 (19.68)	24.20 (29.43)	20.87 (4.62)	5.43 (2.44)	Debargarh (Odisha)
36	IC256547	3	37.00 (26.20)	37.00 (37.45)	17.43 (4.23)	11.70 (3.49)	Debargarh (Odisha)
37	IC256742	3	28.67 (22.50)	28.67 (32.35)	22.97 (4.84)	9.00 (3.08)	Raigad (Chattisgarh)
38	IC256769	3	27.33 (26.50)	27.33 (31.51)	18.30 (4.34)	11.30 (3.43)	Sambalpur (Odisha)
39	IC256780	3	24.83 (23.83)	24.83 (29.84)	16.03 (4.06)	11.10 (3.40)	Sambalpur (Odisha)
40	IC256781	3	38.00 (28.99)	38.00 (38.05)	17.53 (4.24)	16.17 (4.08)	Sambalpur (Odisha)
41	IC267428	3	28.50 (23.12)	28.50 (32.25)	16.57 (4.13)	7.67 (2.86)	Garwal (Bihar)
42	IC273558	3	22.10 (21.27)	22.10 (28.00)	23.63 (4.91)	8.67 (3.03)	Koraput (Odisha)
43	IC274380	3	22.50 (21.77)	22.50 (28.29)	23.67 (4.92)	5.63 (2.48)	Kangra(HimachalPradesh)
44	IC277252	3	25.17 (22.98)	25.17 (30.05)	16.13 (4.08)	7.17 (2.76)	Balasore (Odisha)
45	IC277274	3	35.80 (20.50)	35.80 (36.75)	22.20 (4.76)	4.77 (2.29)	Balasore (Odisha)
46	IC280477	3	33.50 (26.54)	33.50 (35.35)	16.73 (4.15)	11.87 (3.52)	Nayagarh (Odisha)
47	IC280478	3	27.17 (23.86)	27.17 (31.40)	19.87 (4.51)	11.77 (3.47)	Nayagarh (Odisha)
48	IC280556	3	30.17 (27.88)	30.17 (33.22)	16.70 (4.14)	11.50 (3.43)	Nayagarh (Odisha)
49	IC280580	3	24.67 (22.92)	24.67 (29.73)	17.77 (4.25)	8.97 (3.07)	Nayagarh (Odisha)
50	IC280594	3	32.00 (24.06)	32.00 (34.24)	22.07 (4.75)	10.17 (3.20)	Nayagarh (Odisha)
51	IC282420	3	28.50 (22.79)	28.50 (32.25)	19.13 (4.41)	6.70 (2.68)	Palamau (Jharkhand)
52	IC282458	3	34.17 (21.37)	34.17 (35.74)	20.13 (4.53)	7.13 (2.75)	Palamau (Jharkhand)
53	IC282483	3	25.17 (21.94)	25.17 (30.04)	19.10 (4.41)	10.50 (3.31)	Gumla (Jharkhand)
54	IC283010	3	22.50 (23.84)	22.50 (28.29)	18.67 (4.36)	9.07 (3.06)	Cuttack (Odisha)
55	IC283026	3	27.67 (22.60)	27.67 (31.73)	18.37 (4.32)	10.13 (3.19)	Cuttack (Odisha)
56	IC283032	3	33.77 (22.69)	33.77 (35.44)	22.97 (4.84)	6.83 (2.68)	Cuttack (Odisha)
57	IC283041	3	32.03 (24.70)	32.03 (34.45)	16.83 (4.16)	8.40 (2.93)	Cuttack (Odisha)
58	IC283100	3	29.27 (24.20)	29.27 (32.68)	20.70 (4.59)	8.50 (2.97)	Jajpur (Odisha)
59	IC283206	3	28.17 (25.30)	28.17 (31.87)	15.43 (3.99)	8.70 (3.03)	Puri (Odisha)
60	IC283264	3	23.50 (23.82)	23.50 (28.91)	22.30 (4.77)	10.33 (3.29)	Puri (Odisha)
61	IC283277	3	23.67 (25.29)	23.67 (29.07)	23.23 (4.87)	8.93 (3.04)	Puri (Odisha)
62	IC334163	3	24.50 (21.06)	24.50 (29.65)	17.20 (4.21)	12.63 (3.55)	Ranchi (Jharkhand)
63	IC334169	3	28.53 (23.21)	28.53 (32.15)	20.20 (4.53)	6.57 (2.64)	Ranchi (Jharkhand)
64	IC337564	3	35.77 (22.01)	35.77 (36.73)	17.53 (4.24)	9.73 (3.19)	Mirzapur (Uttar Pradesh)
65	IC344686	3	28.33 (23.12)	28.33 (32.15)	18.80 (4.37)	11.20 (3.35)	Bharuch (Gujarat)
66	IC346237	3	29.00 (21.34)	29.00 (32.54)	19.37 (4.44)	8.73 (3.03)	Madhubani (Bihar)
67	IC346258	3	29.67 (25.08)	29.67 (32.83)	18.17 (4.30)	9.30 (3.13)	Vaishali (Bihar)
68	IC410126	3	28.83 (22.72)	28.83 (32.41)	21.20 (4.64)	9.20 (3.02)	Wayanad (Kerala)
69	IC426100	3	22.83 (22.91)	22.83 (28.48)	19.20 (4.42)	7.73 (2.87)	Bastar (Chattisgarh)
70	IC426122	3	24.17 (23.04)	24.17 (29.43)	16.77 (4.15)	6.23 (2.57)	Bastar (Chattisgarh)
71	IC426123	3	27.17 (26.70)	27.17 (31.40)	19.43 (4.45)	9.70 (3.15)	Bastar (Chattisgarh)
72	IC337545	3	30.63 (21.95)	30.63 (33.43)	21.03 (4.62)	8.80 (3.04)	Bhadohi (Uttar Pradesh)
73	IC426136	3	33.83 (23.69)	33.83 (35.55)	21.47 (4.67)	9.23 (3.09)	Dantewada (Chattisgarh)
74	IC514781	3	27.00 (21.06)	27.00 (31.29)	18.47 (4.33)	9.03 (3.08)	Bargarh (Odisha)
75	IC514782	3	33.17 (23.45)	33.17 (35.10)	18.00 (4.29)	10.77 (3.29)	Bargarh (Odisha)
76	IC514792	3	26.67 (20.78)	26.67 (31.01)	20.40 (4.57)	8.37 (2.98)	Bargarh (Odisha)
77	IC514826	3	25.33 (23.24)	25.33 (30.02)	19.03 (4.41)	6.23 (2.57)	Bargarh (Odisha)
78	IC514994	3	27.50 (25.92)	27.50 (31.51)	17.77 (4.26)	7.80 (2.84)	Bargarh (Odisha)
79	IC515125	3	25.33 (21.95)	25.33 (30.15)	19.57 (4.47)	7.77 (2.84)	Bargarh (Odisha)
80	IC515158	3	25.93 (22.24)	25.93 (30.43)	18.97 (4.39)	11.10 (3.40)	Bargarh (Odisha)
81	IC515868	3	31.67 (21.49)	31.67 (34.23)	20.13 (4.54)	13.57 (3.71)	Tamil Nadu
82	IC516005	3	30.43 (22.39)	30.43 (33.44)	15.93 (4.05)	8.63 (3.01)	Samatispur (Bihar)
83	IC517019	3	34.33 (19.95)	34.33 (35.84)	19.50 (4.46)	8.87 (3.06)	Bargarh (Odisha)
84	IC518807	3	27.17 (20.79)	27.17 (31.40)	21.80 (4.72)	5.60 (2.47)	Raipur (Chattisgarh)
85	IC518941	3	23.67 (22.74)	23.67 (29.02)	16.43 (4.11)	6.83 (2.70)	Allahabad (Uttar Pradesh)
86	IC518967	3	29.00 (23.21)	29.00 (32.52)	18.27 (4.30)	7.20 (2.72)	Unknown
87	IC518976	3	32.83 (22.43)	32.83 (34.88)	21.50 (4.68)	11.47 (3.46)	Unknown
88	IC519009	3	29.83 (23.01)	29.83 (33.11)	19.47 (4.45)	14.50 (3.86)	Thrissur (Kerala)
89	IC540340	3	22.17 (23.50)	22.17 (28.05)	16.77 (4.15)	8.73 (3.03)	Ganjam (Odisha)
90	IC558251	3	29.00 (22.22)	29.00 (32.47)	20.53 (4.56)	8.30 (3.03)	Sonitpur (Assam)
91	IC574907	3	25.17 (24.30)	25.17 (30.04)	18.33 (4.32)	7.37 (2.80)	Faizabad (Uttar Pradesh)
92	IC577964	3	31.93 (22.20)	31.93 (34.24)	19.63 (4.48)	7.70 (2.80)	Sylhet, Bangladesh
93	IC578349	3	29.67 (24.42)	29.67 (33.00)	20.87 (4.61)	7.67 (2.84)	Dhaka, Bangladesh
94	IC578748	3	27.27 (20.17)	27.27 (31.32)	19.63 (4.48)	7.13 (2.71)	Karur (Tamil Nadu)
95	IC75751	3	26.83 (22.84)	26.83 (30.97)	19.03 (4.41)	9.57 (3.14)	IIRR (Hyderabad)
96	IC75777	3	28.17 (23.50)	28.17 (32.04)	20.23 (4.55)	7.77 (2.84)	IIRR (Hyderabad)
97	IC75778	3	27.00 (21.16)	27.00 (31.16)	17.20 (4.20)	9.37 (3.11)	IIRR (Hyderabad)
98	IC75792	3	23.83 (25.75)	23.83 (29.13)	20.40 (4.56)	10.93 (3.34)	IIRR (Hyderabad)
99	IC75808	3	25.67 (20.50)	25.67 (30.39)	17.20 (4.21)	7.97 (2.89)	IIRR (Hyderabad)
100	IC75884	3	31.77 (23.96)	31.77 (34.29)	20.43 (4.57)	7.33 (2.77)	IIRR (Hyderabad)
101	IC75887	3	30.60 (23.06)	30.60 (33.54)	18.87 (4.39)	7.77 (2.83)	IIRR (Hyderabad)
102	IC75997	3	24.17 (23.77)	24.17 (29.41)	17.67 (4.26)	8.50 (2.99)	IIRR (Hyderabad)
103	IC256545	3	34.17 (20.78)	34.17 (35.68)	19.20 (4.42)	8.30 (2.94)	Debagarh (Odisha)
104	IC518805	5	63.03 (43.20)	63.03 (52.56)	11.77 (3.50)	13.00 (3.67)	Bargarh (Odisha)
105	IC515974	5	52.43 (41.34)	52.43 (46.40)	14.70 (3.90)	11.57 (3.47)	Unknown
106	IC569465	5	59.63 (37.58)	59.63 (50.55)	15.40 (3.99)	15.43 (3.99)	Meerut (Uttar Pradesh)
107	IC75886	5	55.17 (40.88)	55.17 (47.97)	16.23 (4.09)	15.23 (3.96)	IIRR (Hyderabad)
108	IC200940	5	59.20 (41.07)	59.20 (50.32)	12.20 (3.56)	15.67 (4.02)	Unknown
109	IC518849	5	54.70 (46.05)	54.70 (47.70)	12.03 (3.54)	21.00 (4.64)	Bargarh (Odisha)
110	IC575211	5	52.80 (37.36)	52.80 (46.61)	12.33 (3.58)	17.77 (4.27)	Karimganj (Assam)
111	IC426092	5	51.57 (35.95)	51.57 (45.90)	10.57 (3.33)	15.77 (4.03)	Bastar (Chattisgarh)
112	IC334193	5	58.77 (39.37)	58.77 (50.05)	15.40 (3.99)	15.60 (4.01)	Girdih (Jharkhand)
113	IC346899	5	58.00 (40.68)	58.00 (49.61)	16.23 (4.09)	16.73 (4.14)	Bastar (Chattisgarh)
114	IC256849	5	59.63 (41.26)	59.63 (50.56)	12.20 (3.56)	16.00 (4.06)	Sambalpur (Odisha)
115	IC256842	5	53.27 (40.27)	53.27 (46.87)	12.03 (3.54)	20.67 (4.60)	Sambalpur (Odisha)
116	IC256629	7	71.93 (48.02)	71.93 (58.03)	8.43 (2.99)	13.53 (3.74)	Sambalpur (Odisha)
117	IC517008	7	65.93 (49.12)	65.93 (54.31)	8.63 (3.02)	21.70 (4.71)	Bargarh (Odisha)
118	IC576798	7	77.50 (51.95)	77.50 (61.69)	9.60 (3.18)	26.33 (5.18)	Sedhiou, Senegal
119	IC515511	7	76.10 (48.07)	76.10 (60.76)	9.50 (3.16)	27.63 (5.30)	Cuttack (Odisha)
120	IC75885	7	81.50 (49.06)	81.50 (64.55)	6.63 (2.67)	26.83 (5.23)	IIRR (Hyderabad)
121	IC515838	7	64.73 (53.96)	64.73 (53.59)	6.37 (2.62	30.90 (5.60)	Karnataka
122	IC283249	7	65.93 (51.95)	65.93 (54.31)	9.50 (3.16)	21.70 (4.71)	Puri (Odisha)
123	IC256787	9	87.63 (84.94)	87.63 (69.43)	8.47 (2.99)	51.57 (7.22)	Sambalpur (Odisha)
124	IC277313	9	92.03 (88.09)	92.03 (73.83)	6.57 (2.66)	54.27 (7.40)	Bhadrak (Odisha)
125	IC283007	9	91.27 (88.65)	91.27 (73.16)	7.63 (2.85)	44.77 (6.68)	Cuttack (Odisha)
126	IC283024	9	88.17 (82.22)	88.17 (69.90)	8.77 (3.04)	55.93 (7.51)	Cuttack (Odisha)
127	IC283064	9	82.53 (89.15)	82.53 (65.48)	6.63 (2.67)	47.03 (6.89)	Cuttack (Odisha)
128	IC283087	9	76.60 (88.09)	76.60 (61.09)	7.77 (2.88)	53.77 (7.37)	Cuttack (Odisha)
129	IC283088	9	76.03 (67.21)	76.03 (60.76)	8.77 (3.04)	48.00 (6.96)	Cuttack (Odisha)
130	IC283096	9	90.17 (90.00)	90.17 (71.83)	9.47 (3.16)	55.17 (7.46)	Jajpur (Odisha)
131	IC283105	9	87.17 (89.15)	87.17 (69.11)	9.60 (3.18)	58.53 (7.68)	Jajpur (Odisha)
132	IC283129	9	88.83 (90.00)	88.83 (70.62)	6.73 (2.69)	44.87 (6.73)	Kendrapada (Odisha)
133	IC283245	9	79.97 (90.00)	79.97 (63.43)	9.07 (3.09)	47.87 (6.95)	Puri (Odisha)
134	IC283271	9	74.00 (90.00)	74.00 (59.40)	6.67 (2.68)	57.83 (7.64)	Puri (Odisha)
135	IC283275	9	91.90 (90.00)	91.90 (73.58)	9.77 (3.20)	57.50 (7.62)	Puri (Odisha)
136	IC283294	9	96.60 (90.00)	96.60 (79.93)	8.73 (3.04)	56.87 (7.57)	Puri (Odisha)
137	IC283296	9	95.73 (90.00)	95.73 (78.20)	6.47 (2.64)	55.50 (7.48)	Puri (Odisha)
138	IC326417	9	83.33 (84.00)	83.33 (65.92)	7.60 (2.85)	47.13 (6.90)	Moradabad(UttarPradesh)
139	IC331148	9	83.87 (69.73)	83.87 (66.45)	6.30 (2.61)	53.90 (7.37)	Narmada (Gujarat)
140	IC337530	9	92.23 (90.00)	92.23 (73.97)	8.30 (2.97)	64.10 (8.04)	Varanasi (Uttar Pradesh)
141	IC337563	9	77.50 (90.00)	77.50 (61.70)	9.00 (3.08)	43.90 (6.66)	Mirzapur (Uttar Pradesh)
142	IC340680	9	93.83 (83.82)	93.83 (75.86)	6.57 (2.66)	53.47 (7.35)	Faizabad (Uttar Pradesh)
143	IC340694	9	84.97 (90.00)	84.97 (67.33)	6.67 (2.68)	55.37 (7.47)	Ballia (Uttar Pradesh)
144	IC343386	9	87.23 (80.63)	87.23 (69.10)	7.83 (2.89)	56.23 (7.53)	Wayanad (Kerala)
145	IC343466	9	89.47 (90.00)	89.47 (71.21)	8.77 (3.04)	51.37 (7.20)	Mirzapur (Uttar Pradesh)
146	IC331110	9	96.97 (90.00)	96.97 (80.28)	9.30 (3.13)	52.17 (7.26)	Narmada (Gujarat)
147	IC346206	9	72.83 (90.00)	72.83 (58.60)	6.07 (2.56)	49.33 (7.06)	Darbhanga (Bihar)
148	IC346256	9	77.17 (90.00)	77.17 (61.47)	7.67 (2.86)	56.17 (7.53)	Darbhanga (Bihar)
149	IC346855	9	77.77 (90.00)	77.77 (61.88)	8.50 (3.00)	45.83 (6.81)	Bastar (Chattisgarh)
150	NAVEEN	9	78.40 (90.00)	78.40 (62.31)	9.23 (3.12)	54.20 (7.40)	Cuttack (Odisha)
151	SWARNA	9	88.33 (90.00)	88.33 (70.07)	7.67 (2.86)	45.00 (6.74)	RARS (Andhra Pradesh)
152	TN1	9	94.67 (90.00)	94.67 (77.30)	6.37 (2.62)	53.47 (7.35)	Taiwan
	*P* value		< 0.0001	< 0.0001	< 0.0001	< 0.0001	
	SE(d)		2.63	3.70	2.03	2.02	
	Turkey HSD at 5%		11.9	16.8	9.21	9.2	

**Table 2:** Statistics for standard evaluation system of *N. lugens* resistant scores

Trait	Mean	Standard deviation	Range		Skewness	Kurtosis
			Min	Max		
SES Scores	4.37	2.54	1	9	1	-0.5

### Genotyping

**Fig. 1:**
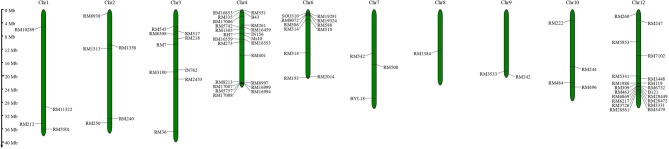
Markers loci position of 82 SSR markers in the rice chromosome used in this study

Genotypes of the panel were assayed using 82 molecular markers associated with 28 resistance genes against *N. lugens* (Fig. 1). Marker assay led to the amplification of 149 alleles (Supplementary Table 3), which consisted of 45 alleles that were the same across all samples (monomorphic) and 104 that varied (polymorphic). Among the markers linked to resistance genes, 37 displayed variability (polymorphism). The number of alleles per marker varied, with a minimum of one and a maximum of eleven, averaging 1.817 alleles per locus. The smallest amplicon measured was 90 base pairs (bp) from marker RM6869, and the largest was 1100 bp from marker RM589. The heterozygosity of the markers (Ho) ranged from 0.00 to 0.25, with an average heterozygosity rate of 0.053. The average frequency of the major allele across the 82 SSR (simple sequence repeat) markers was 0.891, ranging from 0.49 to 0.99 among the 37 polymorphic markers. In contrast, the monomorphic markers were fixed at a frequency of 1.00. The genetic diversity among these markers had a mean of 0.150, with the highest diversity observed at 0.61 for marker RM335 and the lowest at 0.01 for RM17007 among the polymorphic markers. The polymorphism information content (PIC) values for the markers spanned from 0.01 (RM17007) to 0.54 (RM335), with an overall average of 0.128.

### Genetic relatedness through cluster analysis

**Fig. 2:**
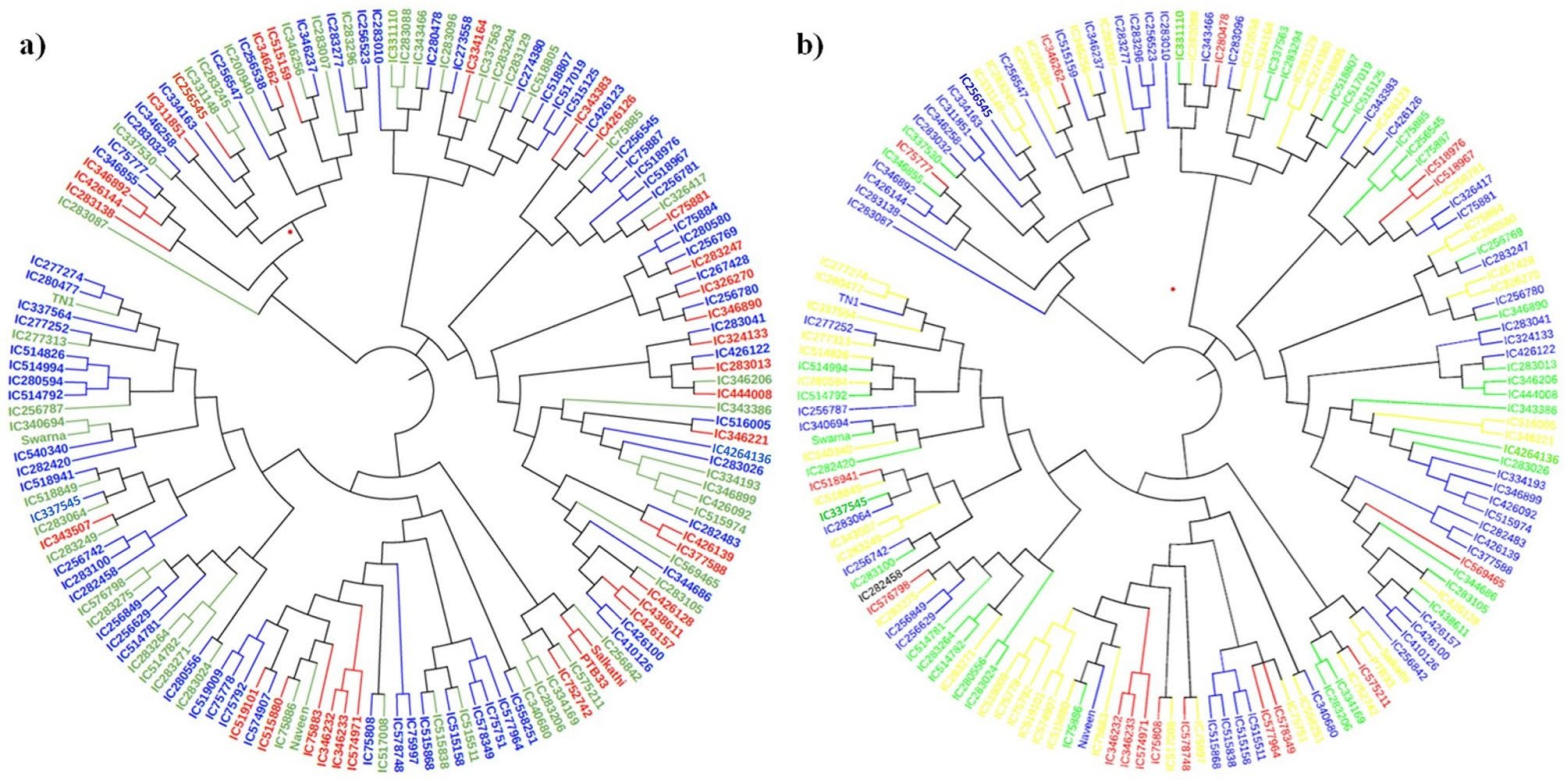
Unrooted neighbor-joining (NJ) tree of 152 rice genotypes based on 82 SSR markers linked to *N. lugens* resistance genes. These genotypes represent (**a**) *N. lugens* resistance reaction (red- resistant; blue-moderately resistant; green- susceptible); (**b**) sub population from structure analysis (red- Sub Population-I; blue – Sub Population-II; green- Sub Population-III; yellow-admixture)

The genetic cluster analysis divided the panel into three principal clusters (I, II, and III) (Fig. 2a), based on their resistance levels to *N. lugens*. These clusters were color-coded to represent different resistance reactions: red for resistant, blue for moderately resistant, and green for susceptible genotypes. Cluster I contained 25 rice genotypes, encompassing 18 resistant or moderately resistant genotypes, 6 genotypes that showed varying levels of susceptibility, and one resistant control genotype (PTB33). Cluster II, which was further divided into two sub-clusters, included a total of 61 genotypes. Sub-cluster II-A comprised 16 genotypes, with 8 exhibiting resistance. On the other hand, sub-cluster II-B had 45 genotypes, 35 of which were resistant and 10 were susceptible. The largest, Cluster III, held 66 genotypes distributed across three sub-clusters (III-A, III-B, and III-C), including 42 resistant or moderately resistant genotypes and 24 displaying various susceptibility levels. Most resistant genotypes were found primarily in cluster III, with a smaller number present in major clusters I and II.

**Fig. 3:**
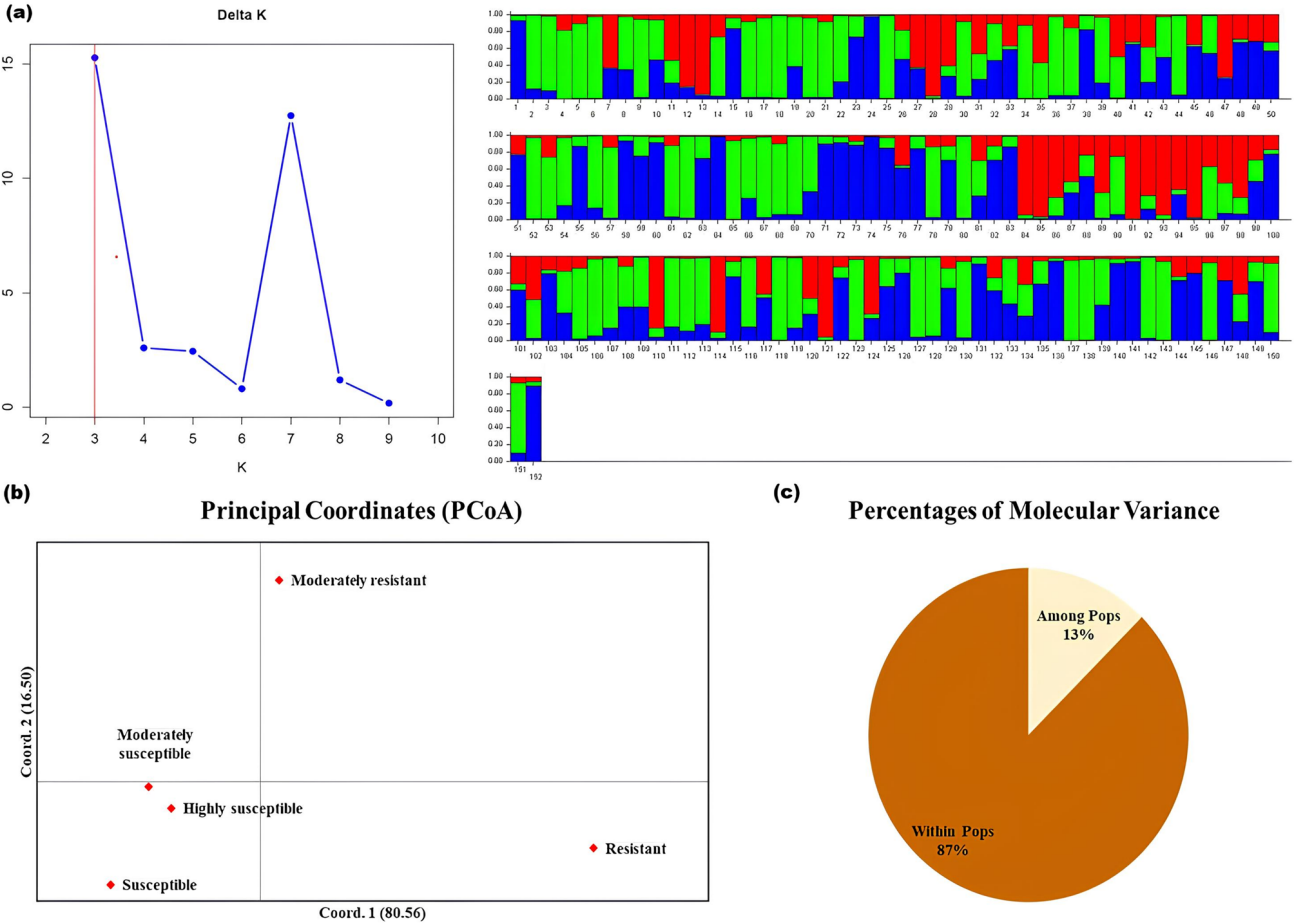
(**a**) Population structure result revealed three subgroups based on coloured segments in the bar plots for ΔK at K = 3; (**b**) Principal Coordinate Analysis and (**c**) Analysis of molecular variance of 152 rice genotypes based on 82 gene linked SSR markers

### Population structure analysis

Population structure analysis revealed that all 152 rice genotypes were optimally grouped into three genetic clusters, achieving a maximum ΔK value of 15.263 at K = 3 (Fig. 3a). Genotypes were classified into sub-populations using a 70% membership probability threshold, with those below this threshold categorized as admixed (Supplementary Table 4). These sub-populations were colour coded as red for Sub-population I (SPI), blue for Sub-population II (SPII), green for Sub-population III (SPIII), and yellow for admixture (AD) (Fig. 2b). SPII, the largest group, comprised 54 genotypes, including 15 resistant, 19 moderately resistant, 6 susceptible, 3 moderately susceptible, and 11 highly susceptible genotypes. SPI contained 15 genotypes, categorized into 3 resistant, 9 moderately resistant, 1 moderately susceptible, and 2 susceptible. The third group, SPIII included 35 genotypes, with 3 resistant, 19 moderately resistant, and a mix of 12 susceptible genotypes. Additionally, the analysis identified 48 genotypes as having mixed ancestry. The analysis was successful in separating resistant from susceptible genotypes into distinct groups. The allele frequency divergence among the three groups, measured as net nucleotide distance, was greatest between SPI and SPIII at 0.069 (Supplementary Table 5). Also, a lower alpha value (α = 0.201) and expected heterozygosity within each sub-population were observed (Supplementary Table 6).

### Principal coordinate (PCoA) analysis

The Principal Coordinates Analysis (PCoA) employing 82 gene-linked markers information showed that the first component (PC1), the second component (PC2), and the third component (PC3) accounted for 80.56%, 16.50% and 2.94% of the variance, respectively, summing up to a total of 100% cumulative variance (Table 3; Fig. 3b). This analysis grouped the moderately susceptible, susceptible, and highly susceptible rice genotypes into one category. In contrast, the moderately resistant and resistant genotypes were segregated into one another, highlighting the genetic distinctions between these groups.

**Table 3:** Genetic diversity of panel population-based PCoA

Axis (%)	1-axis	2-axis	3-axis
Variation of individual axis	80.56	16.50	2.94
Cumulative variation	80.56	97.06	100.00

### AMOVA analysis

Analysis of molecular variance (AMOVA) indicated that 13% of the genetic variation occurred among populations, while 87% was attributed to differences among individuals within populations (Table 4; Fig. 3c). In this study, the fixation index (FST) values across all populations varied between 0 and 1. The highest pairwise FST value, recorded at 0.110, was observed between moderately susceptible and highly susceptible rice genotypes, followed by 0.102 between resistant and moderately susceptible genotypes (Supplementary Table 7).

**Table 4:** AMOVA of the population

Source	df	SS	MS	Est. Var.	%
Among Population	4	2643814.384	660953.596	19830.533	13%
Among individual within Population	147	20341848.563	138379.922	138379.922	87%
Total	151	22985662.947	-	158210.455	100%

### Marker-trait association for *N. lugens* resistance genes

**Fig. 4:**
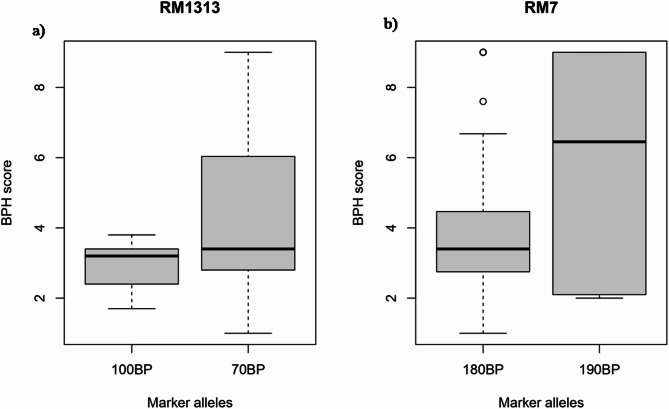
The allelic effects of the associated markers (**a**) RM1313 and (**b**) RM7

In marker-trait association, *p*-value < 0.05% was considered the threshold probability value to identify the significant association in GLM, MLM, and Farm CPU. In the present study, out of 82 molecular markers tested for *N. lugens* resistance, two SSR markers (RM1313 and RM7) were significantly associated with various resistance responses (Table 5). RM1313 marker was standard in GLM, MLM, and Farm CPU analysis. The allelic effects of these markers were plotted against the SES score, where the 100 bp and 180 bp alleles of RM1313 and RM7 markers were associated with resistance against *N. lugens*, respectively (Fig. 4).

**Table 5:** Significant marker-trait associations of tested genotypes against *N. lugens*

Sl. No	Gene	Marker name	Chromosome no.	Position	Model	*P* value	PVE%	Effect
1	*Bph9*	RM1313	2	11,262,954	GLM/MLM/ FarmCPU	0.025/0.021/0.017	6/6/7.2	-1.73/-1.74/-1.74
2	*Qbph3*	RM7	3	10,168,981	FarmCPU	0.04	8%	-1.52

## Discussion

The field populations of *N. lugens* consistently show diverse resistance levels to well-known resistant rice varieties due to evolving behavioral and physiological adaptations. Developing resistant rice varieties remains a vital and eco-friendly pest management strategy. However, the effectiveness of these resistant varieties often diminishes over time as *N. lugens* adapts evolutionarily. Consequently, there is a continuous demand in breeding programs for discovering new resistance genes from diverse rice genetic resources. These discoveries are essential for improving varieties that can withstand the emerging *N. lugens* biotypes, highlighting the importance of tapping into a broad genetic base for long-term resistance. In India, many traditional rice landraces cultivated by local farmers demonstrate adaptability to harsh environmental conditions and hold potential for biotic stress management breeding programs. These traditional rice types exhibit resistance to both biotic and abiotic stresses. Our research corroborates the presence of resistance to *N. lugens* in specific rice genotypes, supporting earlier studies that have identified beneficial genes in landraces and wild relatives for resistance against *N. lugens* (Pati et al. 2018; Babu et al. 2022).

Developing rice varieties resistant to *N. lugens* has proven to be the most cost-effective and environmentally sustainable strategy for controlling this pest. However, these resistant varieties frequently lose their effectiveness due to N. lugens evolutionary adaptation. Consequently, there is a continuous need to identify *N. lugens* resistance genes in new rice sources, which can provide valuable germplasm for breeding programs to create resistant varieties.

In our study, 152 rice genotypes were evaluated and scored (1–9) based on their resistance to *N. lugens*, with 33 genotypes displaying resistance and 70 showing moderate resistance. Among the resistance genotypes, 9 were from Chhattisgarh state. The weather conditions in Chhattisgarh are reported to more favourable for disease establishment, more specifically rain fall (Kumar et al. 2023). This phenomenon triggers co-evolution of resistant and susceptible genotypes at a rapid rate, as per the gene-for-gene hypothesis. Hence, nearly 50% genotypes selected from the Chhattisgarh state showed resistance phenotype and rest 50% susceptible. Rest of origins with relatively more number of genotypes were purely due to large number of genotypes screened from those states. For example, relatively large number of genotypes were screened from Odisha state, hence, resulted in 5 resistant genotype which is not a significant evidence of origin influence. Similarly, Anant et al. (2021) screened 600 varieties from Odisha farmers and identified 18 as resistant and 22 as moderately resistant to *N. lugens*. Pandi et al. (2018) reported 10 resistant red rice varieties out of 240 screened against *N. lugens*. Previous research has also confirmed that using phenotypic parameters to screen rice genotypes for resistance to *N. lugens* is effective for identifying new sources of resistance to address biotype challenges. (Tetarwal et al. 2014; Jena et al. 2015; Kanngan et al. 2023; Meher et al. 2024). Research on feeding marks revealed that resistant genotypes exhibited more feeding marks compared to susceptible ones, suggesting that *N. lugens* may prefer susceptible genotypes. However, this preference was associated with antibiosis or tolerance mechanisms rather than non-preference. Previous studies also supported these findings, showing that *N. lugens* left more feeding marks on resistant genotypes (Anant et al. 2021; Babu et al. 2022). Furthermore, electropenetration graph studies demonstrated that in the susceptible variety TN 1, *N. lugens* could continuously ingest phloem sap without interruption (Ghaffar et al. 2011); suggesting that fewer attempts the insects could settle well for feeding. Whereas, salivation activity did not vary significantly between resistant and susceptible varieties, indicating that *N. lugens* could access the sieve element region in both cases but could only sustain phloem sap ingestion in susceptible genotypes (Ghaffar et al. 2011) and the bug requires more attempt to reach the phloem sap in resistant varieties.

Molecular markers can be used to identify genetic determinants of resistance. Microsatellites, also known as SSR markers, are commonly utilised for genetic diversity studies due to their high polymorphism and informative nature (Vieira et al. 2016; Duan et al. 2017; Babu et al.2022,). In our analysis, all markers exhibited values of average alleles per locus (1.817), PIC (0.128), mean major allele frequency (0.891), and average heterozygosity (0.150). The results suggested that the markers used in the study were informative for genetic analysis. Earlier workers reported similar findings for SSR marker analysis as 104 farmer varieties showed 3.264 alleles per marker against *N. lugens* (Anant et al. 2021). The current study found moderate genetic variation among selected genotypes, consistent with previous results using SSR markers in rice genotypes (Shah et al. 2013; Singh et al. 2013). Moreover, in our study, the PIC value of 0.128, indicated a moderate level of genetic diversity among the analysed genotypes for the resistant loci scattered among genotypes of the panel (Singh et al. 2013). This finding is consistent with Meher et al. (2024), who reported a similar average PIC of 0.137 in their SSR marker study of 191 rice genotypes targeting *N. lugens* resistance. However, our results revealed that 30 genotypes were resistant and 70 were moderately resistant to *N. lugens* out of 152 tested. This demonstrates that despite moderate diversity, some genotypes exhibit strong resistance traits, making them promising subjects for further research and use in managing *N. lugens*.

Cluster analysis revealed three clusters among the rice genotypes investigated. These data demonstrate the effectiveness of SSR markers in separating genotypes into closely related genetic groups, consistent with earlier research (Li et al. 2018; Bhattarai et al. 2021; Khan et al. 2021). Moreover, most of the resistant rice genotypes were grouped under the major cluster III along with the resistant donors PTB33 and Salkathi, indicating genetic similarity and potential shared resistance genes against *N. lugens*. However, even though the rice genotypes originated from the same environments, they were also clustered into distinct clusters, indicating their genetic makeup was different (Ravi et al. 2003). Thus, the differences that were observed could be the result of genotypes that were exposed to and developed under particular environmental conditions, specific geographic locations for genotypes (Jena et al. 2015), as well as the introduction and transfer of rice genotypes from one geographic niche to another (Zhao et al. 2008). Population structure analysis at K = 3 revealed the clear formation of three distinct sub-populations, with some evidence of genetic drift among genotypes. A similar ΔK (K = 3) structure was observed in previous studies involving populations resistant to *N. lugens* (Babu et al. 2022; Meher et al. 2024).

The PCoA analysis revealed genetic variation among the genotypes, as shown by the distinct scatter plots for resistant, moderately resistant, and susceptible groups. Genotypes with similar susceptibility levels clustered together, suggesting genetic similarities. Similarly, resistant and susceptible rice accessions were clustered separately against *N. lugens* (Meher et al. 2024). AMOVA study showed more genetic differentiation between individuals in a population (87%) than between populations (13%).

The marker-trait association analysis revealed that two markers, i.e., RM1313 and RM7, have a significant association with resistance response to *N. lugens.* Among these reported markers, RM7 verified a higher phenotypic variance for percent damage followed by nymphal survival. Previously the RM7 was found to be associated with resistance to *N. lugens* in various rice genotypes (Babu et al. 2022; Meher et al. 2024). The identification of novel R genes and the resistance breeding strategy for creating long-lasting resistant rice varieties would be aided by these reported resistant rice genotypes with resistant markers against *N. lugens*. The PVE of the identified associated markers indicate the portion of the total variation in population explained by the identified gene or QTL. It was true that the population mean score in the current investigation was confounded with relatively high level of standard deviation. However, this level of PVE is not only due to high standard deviation but an indication of complex nature of resistance in the population. On the other hand, the effects of identified association suggest that identified genes reduce the score levels by -1.74 and − 1.52, respectively. The identified marker information may be utilized in marker-assisted resistant breeding programs. Even though the phenotypic variation explained by reported markers was relatively low, these markers can be utilized in genomic prediction-based resistant breeding by considering reported markers as fixed effects in the genomic selection model (Anilkumar et al. 2023). To date, 46 genes resistant to *N. lugens* have been identified, and only a limited number of these genes have been employed in resistant breeding programs (Jena and Kim, 2010; Du et al. 2020). This investigation gathered functional or candidate genes linked to these markers from the rice annotation project database (Supplementary Table 8). Our findings revealed that the marker RM1313, situated on chromosome 2, is associated with the *Bph9* gene, which encodes a unique type of nucleotide-binding and leucine-rich repeat (NLR)-containing protein that is found within the endomembrane system and causes a cell death phenotype. *Bph9* triggers the salicylic acid and jasmonic acid signaling pathways in rice plants, providing resistance through both antixenosis and antibiosis mechanisms against *N. lugens* (Zhao et al. 2016). Similarly, marker RM7, located on chromosome 3 confers resistance against *N. lugens* by antibiosis mechanism (Wei et al. 2009). Hence, the findings have significant scope in utilizing reported markers for resistance breeding programs.

## Conclusion

In this study, 33 rice varieties notably showed higher resistance to *N. lugens*. Furthermore, the investigation revealed the presence of 28 *N. lugens* resistance genes and QTLs within these rice varieties, identified alongside 82 gene-linked markers. The results exhibited that two SSR markers, RM1313 and RM7, associated with Bph9 and Qbph3 *N. lugens* resistance gene, respectively, were significantly linked with all the phenotypic parameters. Consequently, the identified resistant genes from these rice genotypes could be valuable in developing resistant rice varieties against this pest.

## Electronic supplementary material

Below is the link to the electronic supplementary material.

Supplementary Material 1

## Data Availability

The data used in this study are available from the corresponding author on reasonable request.
