# Supplementary material for: Identification of candidate genes for Nilaparvata lugens (stål) resistance through genomic dissection from diverse Indigenous rice genotypes
Source: Bot Stud. 2025 Jul 14;66:20. doi: 10.1186/s40529-025-00461-3 (PMC12259502; doi:10.1186/s40529-025-00461-3)
Supplement: Supplementary file 1 — Supplementary Material 1 [file 40529_2025_461_MOESM1_ESM.docx]

**Supplementary Table 1. Standard Seed-box Screening Technique for *N. lugens* resistance in rice accessions**

| **SCORE** | **RATING*** | **SYMPTOMS** |
| --- | --- | --- |
| 0 | HR | No visible symptom |
| 1 | R | Partially yellowing at first leaf |
| 3 | MR | Partially yellowing first and second leaf |
| 5 | MS | Pronounced yellowing and some wilting |
| 7 | S | Plant severely stunted |
| 9 | HS | All plants dead |
| *HR- Highly Resistance, R- Resistance, MR- Moderately Resistance, MS-Moderately Susceptible, S- Susceptible, HS- Highly Susceptible. | | |

**Supplementary Table 2. List of markers used for genetic association of BPH, *N. lugens* resistance in rice accessions**

| **BPH**  **gene** | **Primer name** | **Chromosome** | **Forward Primer** | **Reverse Primer** | **Expected product size** | **SSR start** | **SSR end** |
| --- | --- | --- | --- | --- | --- | --- | --- |
| *Bph1* | RM 28004 | 12 | GGCTGCCTGCATGGATATATG | ATTATTTCAAGGTCGGAGCCAAGG | 572 | 13114538 | 13115110 |
| *Qbph2* | RM 1358 | 2 | GATCGATGCAGCAGCATATG | ACGTGTGGCTGCTTTTGC | 374 | 10184719 | 10184766 |
|  | RM 28472 | 12 | GCTATGAACCTGTACACATGTAGG | GAGCACCATAACAATCAGTTGC | 331 | 23064571 | 23064600 |
| *Bph3* | RM588 | 6 | GTTGCTCTGCCTCACTCTTG | AACGAGCCAACGAAGCAG | 98 | 1611442 | 1611468 |
| *Qbph3* | RM 19324 | 6 | GAGGTTGTTTGGATGGATAGATGG | AATCCCGTCCTAGAGTTCTTCTACC | 187 | 1675983 | 1676010 |
|  | RM3180 | 3 | GGGTCGGATAGCCACACAC | GAGGTAATCTCGCGGAGTTG | 123 | 18071810 | 18071833 |
|  | RM2453 | 3 | TAGGTGTTCAGGAGTAAAGA | AAACCAGTATTGCTTACAAG | 366 | 20045974 | 20046065 |
|  | RM7 | 3 | TTCGCCATGAAGTCTCTCG | CCTCCCATCATTTCGTTGTT | 180 | 10168981 | 10169358 |
|  | C3-14 | 3 | GGCAAAATTAGACGGCACG | GAATATGCATTTTGTTTGGAG | 250 | 17355957 | 17356196 |
| *Bph4* | RM586 | 6 | ACCTCGCGTTATTAGGTACCC | GAGATACGCCAACGAGATACC | 295 | 1476905 | 1476950 |
|  | RM335 | 4 | GTACACACCCACATCGAGAAGC | TCCATGGATATACGAGGAGATGC | 100 | 679924 | 679959 |
|  | RM401 | 4 | GCATGAGCTGCTCTCATTATTGTCC | GAAACGAACCAAACGTTCATCG | 241 | 13214764 | 13214807 |
| *Bph6* | RM16999 | 4 | GCTGATGCGGAACAAGGAGACC | GATCAGATCACCACCCGAATGAGC | 181 | 21423220 | 21423240 |
|  | RM5757 | 4 | CCTGAGACCATATGCTGCTG | GAGGGAGCATCATTAGCTGG | 130 | 21425181 | 21425204 |
|  | RM17008 | 4 | TTACCTTCGATTAGCTGCTGTTGC | ATTCCTTGCATTACAGACGGTAGC | 174 | 21485304 | 21485355 |
|  | RM314 | 6 | CTAGCAGGAACTCCTTTCAGG | AACATTCCACACACACACGC | 118 | 12566858 | 12567053 |
|  | RM 510 | 6 | GTTTGACGCGATAAACCGACAGC | ATGAGGACGACGAGCAGATTCC | 193 | 2831543 | 2831572 |
|  | RM5742 | 4 | GATCCTCAAACGGCCTCTGC | CCTTCAAAGTTTACTCACGCTCTGC | 192 | 21567979 | 21568002 |
|  | RM119 | 4 | CATCCCCCTGCTGCTGCTGCTG | CGCCGGATGTGTGGGACTAGCG | 166 | 21242569 | 21242734 |
|  | RM6997 | 4 | CGGCAGTAAATTTGCATTGACC | AGTGGCCTTGTCAGTCTACATGC | 152 | 21281309 | 21281344 |
| *Qbph7* | RM542 | 7 | TGAATCAAGCCCCTCACTAC | CTGCAACGAGTAAGGCAGAG | 173 | 12659507 | 12659550 |
|  | RM500 | 7 | GAGCTTGCCAGAGTGGAAAG | GTTACACCGAGAGCCAGCTC | 173 | 15858082 | 15858108 |
|  | RM6217 | 12 | GCAGCAAGAGCAAGAAATCC | GTTCCTGCCGTACCAGCAG | 356 | 22705519 | 22705545 |
| *Bph9* | RM3448 | 12 | CTTCCTCCTTCCTCCTCCTC | CACGTGACACGTACACCCTC | 465 | 19988899 | 19988936 |
|  | RM3533 | 9 | CCTTCATTTCCCTTCCCTCTCC | CTTTCCAACCTGTCAGGGAATCG | 126 | 17833841 | 17833864 |
|  | RM242 | 9 | GGCCAACGTGTGTATGTCTC | TATATGCCAAGACGGATGGG | 265 | 18642201 | 18642222 |
|  | RM1313 | 2 | TGTGTCTGAAAACCAAGGGG | CGTCCAAGCTGTTCGTTCTC | 94 | 11262954 | 11263270 |
|  | RM1986 | 12 | TAACGGAGGGAGTAGTTTTC | GAACCTACATATCGAGAGCA | 286 | 21282462 | 21282499 |
| *Bph10 (t)* | RM484 | 10 | TCTCCCTCCTCACCATTGTC | TGCTGCCCTCTCTCTCTCTC | 299 | 21066729 | 21067027 |
|  | RM496 | 10 | GACATGCGAACAACGACATC | GCTGCGGCGCTGTTATAC | 186 | 21982177 | 21982224 |
| *Bph10* | RM260 | 12 | ACTCCACTATGACCCAGAG | GAACAATCCCTTCTACGATCG | 111 | - | - |
|  | RM5341 | 12 | TGCATTTTCCATACAATACG | ATTTGATACATGGACGATGC | 388 | 19155815 | 19155838 |
|  | RM16459 | 4 | TCCAGGAGTTTGCCTTGTAGTGC | TAGCGAAGTCAGGATGGCATAGG | 191 | 5178652 | 5178699 |
| *Bph12* | RM1305 | 4 | ACCTGCATCGTATGTGTGTG | TCTGGGGTAAACACTGGGAG | 150 | 5624467 | 5624490 |
|  | RM545 | 3 | CAATGGCAGAGACCCAAAAG | CTGGCATGTAACGACAGTGG | 166 | 4916484 | 4916543 |
| *Bph13* | RM517 | 3 | GGCTTACTGGCTTCGATTTG | CGTCTCCTTTGGTTAGTGCC | 190 | 6134606 | 6134635 |
|  | RM250 | 2 | GGTTCAAACCAAGCTGATCA | GATGAAGGCCTTCCACGCAG | 153 | 32774365 | 32774553 |
|  | RM240 | 2 | CCTTAATGGGTAGTGTGCAC | TGTAACCATTCCTTCCATCC | 132 | 31497147 | 31497256 |
|  | RM261 | 4 | CTACTTCTCCCCTTGTGTCG | TGTACCATCGCCAAATCTCC | 125 | 6574396 | 6574518 |
| *Bph14* | IN762 | 3 | CTGCTGCTGCTCTCGTATTG | CAGGGAAGCTCCAAGAACAG | 170 | 17381292 | 17381462 |
| *Bph15* | RM6732 | 12 | AATTTTGAACACCTCAAAGG | TTTTCAGTGCATGTCTTCG | 196 | 22016908 | 22016967 |
|  | RM20M14 | 12 | ATGCTGACGGTGCTAGGAGT | CAGTCCATCCACACAACTTGA | 140 | 970538 | 970958 |
|  | IN156 | 4 | AGGTGAAGCTGATGTGCTTG | CGATACTTATTGCAACACAC | 180 | 6774842 | 6775023 |
| *Bph16* | RM10289 | 1 | CTTGATTGGCTCTTCTGTCAATGG | GAATTCGATCTGCATCTGTCACG | 89 | 4989087 | 4989112 |
|  | RM8213 | 4 | AGCCCAGTGATACAAAGATG | GCGAGGAGATACCAAGAAAG | 179 | 4418222 | 4418259 |
| *Bph17* | RM28449 | 12 | CACCCATTGATGTGAAACTCTGG | GGATTCATGATACAGTGTGCAACG | 346 | 22689921 | 22689942 |
|  | RH7 | 4 | CTTGCGTTCCGTAGGAGAAG | TGAGTGTAACCCGAAGTGGC | 180 | 6776228 | 6776406 |
| *Bph18* | RM3331 | 12 | CCTCCTCCATGAGCTAATGC | AGGAGGAGCGGATTTCTCTC | 174 | 23528087 | 23528116 |
|  | RM273 | 4 | GAAGCCGTCGTGAAGTTACC | GTTTCCTACCTGATCGCGAC | 207 | 20853335 | 20853691 |
|  | RM36 | 3 | CAACTATGCACCATTGTCGC | GTACTCCACAAGACCGTACC | 192 | 35316162 | 35316253 |
| *Bph19* | RM6308 | 3 | TCGACCTGGCTCTCCTCTAG | TATCAACCTGCTCCTCCTGG | 166 | 7148679 | 7148705 |
|  | RM16559 | 4 | CCTGGAACCTGGAGGTGTTCTCG | GTCGTGGACGATTTCTTCGTCAGC | 198 | 9404151 | 9404171 |
|  | RM218 | 3 | TGGTCAAACCAAGGTCCTTC | GACATACATTCTACCCCCGG | 148 | 8405368 | 8405516 |
| *Bph20* | RM16553 | 4 | CATAGCCACTTATCGTTGTTACGC | TGTCCATCTATGACTGTCCACTACG | 182 | 8601616 | 8601643 |
|  | B43 | 4 | ACTCCAATTGGTTCCTGTGG | TGGACTAAAAGCCGATGAGC | 764 | 720187 | 720950 |
|  | RM3726 | 12 | CACACACATCGCTCGGTC | GATGTGGAGGTCGATGGC | 193 | 23308950 | 23308981 |
| *Bph20(t)* | RM5953 | 4 | AAACTTTCTGTGATGGTATC | ATCCTTGTCTAGAATTGACA | 129 | 9379510 | 9379636 |
| *Bph21* | B121 | 12 | CGTCGTACATTCTGAAATGGAG | GGACATGGAGATGGTGGAGA | 101 | 22283711 | 22283797 |
|  | RM28561 | 12 | CTTCAAGACTGGCCCAATATTACTGC | TGACTGAAGCCTTCTTCACTTGC | 260 | 24117220 | 24117251 |
| *Bph25* | SOO310 | 6 | CAACAAGATGGACGGCAAGG | TTGGAAGAAAAGGCAGGCAC | 215 | 102771 | 103001 |
|  | RM5479 | 12 | AACTCCTGATGCCTCCTAAG | TCCATAGAAACAATTTGTGC | 292 | 24446205 | 24446246 |
| *Bph26* | MS10 | 4 | CAATACGAGAAGCCCCTCAC | CTGAAGGAACACGCGGTAGT | 167 | 7350878 | 7350759 |
|  | RM309 | 12 | GTAGATCACGCACCTTTCTGG | AGAAGGCCTCCGGTGAAG | 178 | 21521910 | 21521937 |
| *Bph28* | RM16853 | 4 | CTCCCATCCTTCATTTCATCTCG | CTTTCTGCAAGACACTGCAAACG | 170 | 19212712 | 19212731 |
| *Bph29* | BYL18 | 6 | CCCACTTCCACAACCACA | ATGCTCCTAGCTTCCTATTCC | 180 | 324792 | 324973 |
|  | RM244 | 10 | CCGACTGTTCGTCCTTATCA | CTGCTCTCGGGTGAACGT | 163 | 16497421 | 16497901 |
| *Bph30* | RM222 | 10 | CTTAAATGGGCCACATGCG | CAAAGCTTCCGGCCAAAAG | 213 | 3128793 | 3129106 |
|  | RM19291 | 6 | CACTTGCACGTGTCCTCTGTACG | GTGTTTCAGTTCACCTTGCATCG | 147 | 1215950 | 1215977 |
|  | RM 193 | 6 | CGTGAGGGAGGACTACTGCTC | GATGATGCTGTAGAGCCTTGTG | 189 | 18085431 | 18,085,619 |
| *Bph31* | RM8072 | 6 | GATCACTCAGGTCATCCATTC | AATCAGAGAGGCTAAAGACAATAAT | 146 | 1408336 | 1408481 |
| *Bph32* | RM11522 | 1 | TAACTGCAGTGCTCAACAAAGG | CTAGGTACCGGATTAAGATTCACC | 376 | 28065132 | 28065182 |
| *Bph33* | RM17006 | 4 | AGTCGACGAAGAGGTAGTCGATGG | CTCCTCCTGCTGCTCTCCTTCC | 136 | 21473220 | 21473240 |
|  | RM 6938 | 2 | CTCCGCGGAGTCGATTTTA | AACCAACCACTGATTATTCCAACT | 297 | 977799 | 977912 |
|  | RM1384 | 8 | TTAATCCATCCTGTAGCTGG | TCGCTATCAACACTACCTGC | 360 | 11842678 | 11842749 |
|  | RM551 | 4 | AGCCCAGACTAGCATGATTG | GAAGGCGAGAAGGATCACAG | 186 | 168711 | 168746 |
|  | RM5501 | 1 | GCGCTTCTACTTCCACAAGG | GGTTGGCGTACGTAGAGAGG | 155 | 34542232 | 34542285 |
|  | RM212 | 1 | CCACTTTCAGCTACTACCAG | CACCCATTTGTCTCTCATTATG | 136 | 33053493 | 33053654 |
|  | RM16994 | 4 | TGGCAGTACACACTACAGTACATGC | AGAGGGAGGAGAGAAAGGAAGG | 239 | 21331444 | 21331464 |
| *Bph34* | RM17007 | 4 | AGTCGACGAAGAGGTAGTCGATGG | CTCCTCCTGCTGCTCTCCTTCC | 136 | 21473220 | 21473240 |

**Supplementary Table 3. Genetic diversity indices and amplification pattern of 82 marker loci used for rice genotypes against *N. lugens***

| **Marker used** | **Major Allele Frequency** | **Genotype No** | **Allele No** | **Gene Diversity** | **Heterozygosity** | **PIC** |
| --- | --- | --- | --- | --- | --- | --- |
| RM247 | 0.80 | 3.00 | 3.00 | 0.33 | 0.00 | 0.30 |
| RM7102 | 1.00 | 1.00 | 1.00 | 0.00 | 0.00 | 0.00 |
| RM6869 | 0.61 | 11.00 | 11.00 | 0.52 | 0.09 | 0.43 |
| RM463 | 1.00 | 1.00 | 1.00 | 0.00 | 0.00 | 0.00 |
| RM1358 | 0.50 | 1.00 | 2.00 | 0.50 | 1.00 | 0.38 |
| RM28472 | 1.00 | 1.00 | 1.00 | 0.00 | 0.00 | 0.00 |
| RM588 | 1.00 | 1.00 | 1.00 | 0.00 | 0.00 | 0.00 |
| RM19324 | 1.00 | 1.00 | 1.00 | 0.00 | 0.00 | 0.00 |
| RM3180 | 1.00 | 1.00 | 1.00 | 0.00 | 0.00 | 0.00 |
| RM2453 | 1.00 | 1.00 | 1.00 | 0.00 | 0.00 | 0.00 |
| RM7 | 0.89 | 3.00 | 2.00 | 0.19 | 0.06 | 0.17 |
| C-314 | 1.00 | 1.00 | 1.00 | 0.00 | 0.00 | 0.00 |
| RM586 | 0.83 | 3.00 | 2.00 | 0.29 | 0.19 | 0.25 |
| RM335 | 0.49 | 4.00 | 3.00 | 0.61 | 0.14 | 0.54 |
| RM401 | 0.76 | 2.00 | 2.00 | 0.37 | 0.00 | 0.30 |
| RM119 | 1.00 | 1.00 | 1.00 | 0.00 | 0.00 | 0.00 |
| RM16999 | 1.00 | 1.00 | 1.00 | 0.00 | 0.00 | 0.00 |
| RM5757 | 0.71 | 3.00 | 3.00 | 0.42 | 0.00 | 0.34 |
| RM17008 | 0.63 | 3.00 | 3.00 | 0.54 | 0.00 | 0.48 |
| RM314 | 1.00 | 1.00 | 1.00 | 0.00 | 0.00 | 0.00 |
| RM510 | 1.00 | 1.00 | 1.00 | 0.00 | 0.00 | 0.00 |
| RM6997 | 0.80 | 3.00 | 3.00 | 0.35 | 0.00 | 0.32 |
| RM5742 | 1.00 | 1.00 | 1.00 | 0.00 | 0.00 | 0.00 |
| RM542 | 1.00 | 1.00 | 1.00 | 0.00 | 0.00 | 0.00 |
| RM500 | 0.91 | 4.00 | 4.00 | 0.16 | 0.00 | 0.15 |
| RM6217 | 0.89 | 3.00 | 2.00 | 0.19 | 0.11 | 0.17 |
| RM3448 | 0.50 | 2.00 | 3.00 | 0.53 | 1.00 | 0.42 |
| RM1313 | 0.50 | 1.00 | 2.00 | 0.50 | 1.00 | 0.38 |
| RM1986 | 0.62 | 4.00 | 3.00 | 0.47 | 0.26 | 0.36 |
| RM3533 | 0.78 | 4.00 | 3.00 | 0.36 | 0.25 | 0.33 |
| RM242 | 1.00 | 1.00 | 1.00 | 0.00 | 0.00 | 0.00 |
| RM260 | 0.67 | 4.00 | 3.00 | 0.47 | 0.01 | 0.40 |
| RM484 | 1.00 | 1.00 | 1.00 | 0.00 | 0.00 | 0.00 |
| RM5341 | 1.00 | 1.00 | 1.00 | 0.00 | 0.00 | 0.00 |
| RM496 | 0.94 | 2.00 | 2.00 | 0.11 | 0.00 | 0.11 |
| RM16459 | 0.84 | 3.00 | 3.00 | 0.28 | 0.00 | 0.26 |
| RM1305 | 0.52 | 3.00 | 2.00 | 0.50 | 0.01 | 0.37 |
| RM545 | 1.00 | 1.00 | 1.00 | 0.00 | 0.00 | 0.00 |
| RM517 | 1.00 | 1.00 | 1.00 | 0.00 | 0.00 | 0.00 |
| RM250 | 1.00 | 1.00 | 1.00 | 0.00 | 0.00 | 0.00 |
| RM240 | 0.93 | 2.00 | 2.00 | 0.12 | 0.00 | 0.12 |
| RM261 | 1.00 | 1.00 | 1.00 | 0.00 | 0.00 | 0.00 |
| IN762 | 1.00 | 1.00 | 1.00 | 0.00 | 0.00 | 0.00 |
| RM6732 | 1.00 | 1.00 | 1.00 | 0.00 | 0.00 | 0.00 |
| IN156 | 1.00 | 1.00 | 1.00 | 0.00 | 0.00 | 0.00 |
| RM20M14 | 1.00 | 1.00 | 1.00 | 0.00 | 0.00 | 0.00 |
| RM10289 | 1.00 | 1.00 | 1.00 | 0.00 | 0.00 | 0.00 |
| RM8213 | 0.64 | 3.00 | 3.00 | 0.49 | 0.00 | 0.40 |
| RH7 | 1.00 | 1.00 | 1.00 | 0.00 | 0.00 | 0.00 |
| RM28449 | 0.76 | 2.00 | 2.00 | 0.37 | 0.00 | 0.30 |
| RM3331 | 1.00 | 1.00 | 1.00 | 0.00 | 0.00 | 0.00 |
| RM273 | 1.00 | 1.00 | 1.00 | 0.00 | 0.00 | 0.00 |
| RM36 | 0.74 | 5.00 | 5.00 | 0.42 | 0.00 | 0.39 |
| RM6308 | 1.00 | 1.00 | 1.00 | 0.00 | 0.00 | 0.00 |
| RM16559 | 1.00 | 1.00 | 1.00 | 0.00 | 0.00 | 0.00 |
| RM218 | 0.82 | 2.00 | 2.00 | 0.29 | 0.00 | 0.25 |
| RM16553 | 1.00 | 1.00 | 1.00 | 0.00 | 0.00 | 0.00 |
| B43 | 1.00 | 1.00 | 1.00 | 0.00 | 0.00 | 0.00 |
| RM5953 | 1.00 | 1.00 | 1.00 | 0.00 | 0.00 | 0.00 |
| RM3726 | 1.00 | 1.00 | 1.00 | 0.00 | 0.00 | 0.00 |
| B121 | 1.00 | 1.00 | 1.00 | 0.00 | 0.00 | 0.00 |
| RM28561 | 0.76 | 4.00 | 4.00 | 0.39 | 0.00 | 0.36 |
| SOO310 | 0.89 | 2.00 | 2.00 | 0.20 | 0.00 | 0.18 |
| RM5479 | 0.59 | 3.00 | 3.00 | 0.52 | 0.00 | 0.42 |
| Ms10 | 1.00 | 1.00 | 1.00 | 0.00 | 0.00 | 0.00 |
| RM309 | 1.00 | 1.00 | 1.00 | 0.00 | 0.00 | 0.00 |
| RM16853 | 0.96 | 3.00 | 2.00 | 0.08 | 0.01 | 0.07 |
| BYL18 | 1.00 | 1.00 | 1.00 | 0.00 | 0.00 | 0.00 |
| RM244 | 1.00 | 1.00 | 1.00 | 0.00 | 0.00 | 0.00 |
| RM222 | 0.84 | 4.00 | 3.00 | 0.28 | 0.03 | 0.25 |
| RM19291 | 0.52 | 4.00 | 3.00 | 0.51 | 0.05 | 0.39 |
| RM8072 | 0.82 | 2.00 | 2.00 | 0.29 | 0.00 | 0.25 |
| RM193 | 0.91 | 2.00 | 2.00 | 0.17 | 0.00 | 0.15 |
| RM11522 | 1.00 | 1.00 | 1.00 | 0.00 | 0.00 | 0.00 |
| RM6938 | 1.00 | 1.00 | 1.00 | 0.00 | 0.00 | 0.00 |
| RM1384 | 0.95 | 2.00 | 2.00 | 0.10 | 0.11 | 0.09 |
| RM551 | 0.84 | 2.00 | 2.00 | 0.27 | 0.00 | 0.24 |
| RM5501 | 0.93 | 2.00 | 2.00 | 0.12 | 0.00 | 0.12 |
| RM212 | 1.00 | 1.00 | 1.00 | 0.00 | 0.00 | 0.00 |
| RM16994 | 1.00 | 1.00 | 1.00 | 0.00 | 0.00 | 0.00 |
| RM17006 | 1.00 | 1.00 | 1.00 | 0.00 | 0.00 | 0.00 |
| RM17007 | 0.99 | 2.00 | 2.00 | 0.01 | 0.00 | 0.01 |
| **Mean** | **0.891** | **1.915** | **1.817** | **0.150** | **0.053** | **0.128** |

**PIC: Polymorphic information content**

**Supplementary Table 4. Population structure groups of rice genotypes based on inferred ancestry values**

| **Sl. No.** | **Rice genotypes** | **Q1** | **Q2** | **Q3** | **Sub population** | **Resistant reaction** |
| --- | --- | --- | --- | --- | --- | --- |
| 1 | IC283013 | 0.008 | 0.061 | 0.932 | SPIII | R |
| 2 | IC283138 | 0.007 | 0.871 | 0.122 | SPII | R |
| 3 | IC283247 | 0.015 | 0.879 | 0.106 | SPII | R |
| 4 | IC311851 | 0.182 | 0.813 | 0.005 | SPII | R |
| 5 | IC322922 | 0.105 | 0.883 | 0.012 | SPII | R |
| 6 | IC324133 | 0.026 | 0.965 | 0.010 | SPII | R |
| 7 | IC326270 | 0.622 | 0.015 | 0.362 | AD | R |
| 8 | IC334164 | 0.027 | 0.623 | 0.350 | AD | R |
| 9 | IC343383 | 0.055 | 0.917 | 0.029 | SPII | R |
| 10 | IC343507 | 0.064 | 0.465 | 0.470 | AD | R |
| 11 | IC346221 | 0.536 | 0.270 | 0.194 | AD | R |
| 12 | IC346232 | 0.849 | 0.015 | 0.137 | SPI | R |
| 13 | IC346233 | 0.942 | 0.013 | 0.045 | SPI | R |
| 14 | IC346262 | 0.262 | 0.700 | 0.038 | SPII | R |
| 15 | IC346890 | 0.038 | 0.124 | 0.838 | SPIII | R |
| 16 | IC346892 | 0.082 | 0.892 | 0.026 | SPII | R |
| 17 | IC377588 | 0.037 | 0.939 | 0.024 | SPII | R |
| 18 | IC426126 | 0.013 | 0.968 | 0.019 | SPII | R |
| 19 | IC426128 | 0.023 | 0.588 | 0.389 | AD | R |
| 20 | IC426139 | 0.040 | 0.937 | 0.024 | SPII | R |
| 21 | IC426144 | 0.083 | 0.889 | 0.028 | SPII | R |
| 22 | IC426157 | 0.008 | 0.780 | 0.212 | SPII | R |
| 23 | IC438611 | 0.018 | 0.242 | 0.740 | SPIII | R |
| 24 | IC444008 | 0.009 | 0.019 | 0.971 | SPIII | R |
| 25 | IC515159 | 0.011 | 0.981 | 0.009 | SPII | R |
| 26 | IC515880 | 0.183 | 0.345 | 0.473 | AD | R |
| 27 | IC519101 | 0.622 | 0.017 | 0.360 | AD | R |
| 28 | IC574971 | 0.958 | 0.029 | 0.014 | SPI | R |
| 29 | IC752742 | 0.606 | 0.117 | 0.276 | AD | R |
| 30 | IC75881 | 0.080 | 0.883 | 0.037 | SPII | R |
| 31 | IC75883 | 0.461 | 0.302 | 0.237 | AD | R |
| 32 | PTB33 | 0.104 | 0.433 | 0.464 | AD | R |
| 33 | Salkathi | 0.365 | 0.046 | 0.589 | AD | R |
| 34 | IC256523 | 0.125 | 0.863 | 0.012 | SPII | MR |
| 35 | IC256538 | 0.570 | 0.422 | 0.007 | AD | MR |
| 36 | IC256547 | 0.026 | 0.930 | 0.044 | SPII | MR |
| 37 | IC256742 | 0.154 | 0.800 | 0.046 | SPII | MR |
| 38 | IC256769 | 0.011 | 0.167 | 0.822 | SPIII | MR |
| 39 | IC256780 | 0.029 | 0.776 | 0.194 | SPII | MR |
| 40 | IC256781 | 0.500 | 0.479 | 0.022 | AD | MR |
| 41 | IC267428 | 0.324 | 0.019 | 0.656 | AD | MR |
| 42 | IC273558 | 0.381 | 0.412 | 0.207 | AD | MR |
| 43 | IC274380 | 0.028 | 0.474 | 0.498 | AD | MR |
| 44 | IC277252 | 0.009 | 0.940 | 0.051 | SPII | MR |
| 45 | IC277274 | 0.357 | 0.016 | 0.627 | AD | MR |
| 46 | IC280477 | 0.011 | 0.446 | 0.543 | AD | MR |
| 47 | IC280478 | 0.742 | 0.015 | 0.243 | SPI | MR |
| 48 | IC280556 | 0.290 | 0.034 | 0.676 | AD | MR |
| 49 | IC280580 | 0.312 | 0.006 | 0.682 | AD | MR |
| 50 | IC280594 | 0.326 | 0.096 | 0.578 | AD | MR |
| 51 | IC282420 | 0.216 | 0.017 | 0.768 | SPIII | MR |
| 52 | IC282458 | 0.028 | 0.956 | 0.017 | SPII | MR |
| 53 | IC282483 | 0.252 | 0.727 | 0.021 | SPII | MR |
| 54 | IC283010 | 0.028 | 0.794 | 0.178 | SPII | MR |
| 55 | IC283026 | 0.014 | 0.111 | 0.876 | SPIII | MR |
| 56 | IC283032 | 0.007 | 0.849 | 0.145 | SPII | MR |
| 57 | IC283041 | 0.139 | 0.837 | 0.024 | SPII | MR |
| 58 | IC283100 | 0.007 | 0.057 | 0.936 | SPIII | MR |
| 59 | IC283206 | 0.013 | 0.228 | 0.759 | SPIII | MR |
| 60 | IC283264 | 0.017 | 0.062 | 0.921 | SPIII | MR |
| 61 | IC283277 | 0.119 | 0.843 | 0.038 | SPII | MR |
| 62 | IC334163 | 0.011 | 0.964 | 0.024 | SPII | MR |
| 63 | IC334169 | 0.013 | 0.252 | 0.735 | SPIII | MR |
| 64 | IC344686 | 0.010 | 0.010 | 0.980 | SPIII | MR |
| 65 | IC346237 | 0.061 | 0.926 | 0.013 | SPII | MR |
| 66 | IC346258 | 0.034 | 0.708 | 0.258 | SPII | MR |
| 67 | IC410126 | 0.017 | 0.949 | 0.034 | SPII | MR |
| 68 | IC426100 | 0.098 | 0.836 | 0.066 | SPII | MR |
| 69 | IC426122 | 0.013 | 0.917 | 0.071 | SPII | MR |
| 70 | IC426123 | 0.009 | 0.649 | 0.341 | AD | MR |
| 71 | IC499807 | 0.010 | 0.087 | 0.903 | SPIII | MR |
| 72 | IC499813 | 0.019 | 0.060 | 0.921 | SPIII | MR |
| 73 | IC514781 | 0.071 | 0.038 | 0.891 | SPIII | MR |
| 74 | IC514782 | 0.008 | 0.008 | 0.984 | SPIII | MR |
| 75 | IC514792 | 0.028 | 0.122 | 0.850 | SPIII | MR |
| 76 | IC514826 | 0.356 | 0.029 | 0.615 | AD | MR |
| 77 | IC514994 | 0.014 | 0.138 | 0.849 | SPIII | MR |
| 78 | IC515158 | 0.130 | 0.841 | 0.029 | SPII | MR |
| 79 | IC515125 | 0.122 | 0.169 | 0.709 | SPIII | MR |
| 80 | IC515868 | 0.009 | 0.965 | 0.026 | SPII | MR |
| 81 | IC516005 | 0.300 | 0.412 | 0.288 | AD | MR |
| 82 | IC517019 | 0.126 | 0.166 | 0.708 | SPIII | MR |
| 83 | IC518807 | 0.014 | 0.117 | 0.869 | SPIII | MR |
| 84 | IC518941 | 0.937 | 0.042 | 0.020 | SPI | MR |
| 85 | IC518967 | 0.963 | 0.018 | 0.020 | SPI | MR |
| 86 | IC518976 | 0.735 | 0.215 | 0.050 | SPI | MR |
| 87 | IC519009 | 0.546 | 0.128 | 0.326 | AD | MR |
| 88 | IC540340 | 0.229 | 0.252 | 0.519 | AD | MR |
| 89 | IC558251 | 0.674 | 0.304 | 0.021 | AD | MR |
| 90 | IC574907 | 0.249 | 0.686 | 0.066 | AD | MR |
| 91 | IC577964 | 0.985 | 0.010 | 0.005 | SPI | MR |
| 92 | IC578349 | 0.714 | 0.153 | 0.134 | SPI | MR |
| 93 | IC578748 | 0.936 | 0.053 | 0.011 | SPI | MR |
| 94 | IC75751 | 0.639 | 0.059 | 0.302 | AD | MR |
| 95 | IC75777 | 0.969 | 0.016 | 0.015 | SPI | MR |
| 96 | IC75778 | 0.369 | 0.617 | 0.014 | AD | MR |
| 97 | IC75792 | 0.561 | 0.357 | 0.082 | AD | MR |
| 98 | IC75808 | 0.734 | 0.190 | 0.076 | SPI | MR |
| 99 | IC75884 | 0.287 | 0.251 | 0.463 | AD | MR |
| 100 | IC75887 | 0.171 | 0.049 | 0.780 | SPIII | MR |
| 101 | IC75997 | 0.327 | 0.069 | 0.604 | AD | MR |
| 102 | IC337564 | 0.511 | 0.460 | 0.029 | AD | MR |
| 103 | IC256545 | 0.162 | 0.043 | 0.796 | SPIII | MR |
| 104 | IC200940 | 0.176 | 0.494 | 0.330 | AD | S |
| 105 | IC256842 | 0.136 | 0.836 | 0.028 | SPII | S |
| 106 | IC256849 | 0.031 | 0.910 | 0.059 | SPII | S |
| 107 | IC515974 | 0.018 | 0.832 | 0.151 | SPII | S |
| 108 | IC518805 | 0.119 | 0.476 | 0.405 | AD | S |
| 109 | IC518849 | 0.009 | 0.586 | 0.405 | AD | S |
| 110 | IC569465 | 0.845 | 0.112 | 0.044 | SPI | S |
| 111 | IC426092 | 0.020 | 0.812 | 0.168 | SPII | S |
| 112 | IC334193 | 0.028 | 0.855 | 0.117 | SPII | S |
| 113 | IC346899 | 0.020 | 0.786 | 0.194 | SPII | S |
| 114 | IC575211 | 0.897 | 0.074 | 0.029 | SPI | S |
| 115 | IC75886 | 0.063 | 0.174 | 0.763 | SPIII | S |
| 116 | IC256629 | 0.015 | 0.816 | 0.170 | SPII | MS |
| 117 | IC283249 | 0.448 | 0.042 | 0.509 | AD | MS |
| 118 | IC515511 | 0.014 | 0.973 | 0.013 | SPII | MS |
| 119 | IC515838 | 0.021 | 0.828 | 0.151 | SPII | MS |
| 120 | IC517008 | 0.499 | 0.184 | 0.317 | AD | MS |
| 121 | IC576798 | 0.956 | 0.036 | 0.008 | SPI | MS |
| 122 | IC75885 | 0.127 | 0.130 | 0.743 | SPIII | MS |
| 123 | IC256787 | 0.038 | 0.950 | 0.011 | SPII | HS |
| 124 | IC277313 | 0.680 | 0.052 | 0.268 | AD | HS |
| 125 | IC283007 | 0.022 | 0.328 | 0.650 | AD | HS |
| 126 | IC283024 | 0.023 | 0.177 | 0.800 | SPIII | HS |
| 127 | IC283064 | 0.010 | 0.943 | 0.048 | SPII | HS |
| 128 | IC283087 | 0.008 | 0.934 | 0.059 | SPII | HS |
| 129 | IC283088 | 0.138 | 0.240 | 0.622 | AD | HS |
| 130 | IC283096 | 0.058 | 0.906 | 0.037 | SPII | HS |
| 131 | IC283105 | 0.011 | 0.081 | 0.908 | SPIII | HS |
| 132 | IC283129 | 0.253 | 0.149 | 0.599 | AD | HS |
| 133 | IC283245 | 0.027 | 0.533 | 0.440 | AD | HS |
| 134 | IC283271 | 0.329 | 0.372 | 0.299 | AD | HS |
| 135 | IC283275 | 0.055 | 0.272 | 0.673 | AD | HS |
| 136 | IC283294 | 0.023 | 0.030 | 0.947 | SPIII | HS |
| 137 | IC283296 | 0.050 | 0.943 | 0.008 | SPII | HS |
| 138 | IC326417 | 0.038 | 0.949 | 0.013 | SPII | HS |
| 139 | IC331148 | 0.025 | 0.549 | 0.426 | AD | HS |
| 140 | IC337530 | 0.033 | 0.049 | 0.918 | SPIII | HS |
| 141 | IC337563 | 0.028 | 0.036 | 0.936 | SPIII | HS |
| 142 | IC340680 | 0.014 | 0.953 | 0.033 | SPII | HS |
| 143 | IC340694 | 0.058 | 0.928 | 0.014 | SPII | HS |
| 144 | IC343386 | 0.239 | 0.046 | 0.715 | SPIII | HS |
| 145 | IC343642 | 0.194 | 0.009 | 0.797 | SPIII | HS |
| 146 | IC343466 | 0.075 | 0.911 | 0.014 | SPII | HS |
| 147 | IC346206 | 0.284 | 0.008 | 0.708 | SPIII | HS |
| 148 | IC346256 | 0.444 | 0.323 | 0.233 | AD | HS |
| 149 | IC346855 | 0.065 | 0.234 | 0.700 | SPIII | HS |
| 150 | **TN1** | 0.082 | 0.812 | 0.106 | SPII | HS |
| 151 | **Naveen** | 0.069 | 0.829 | 0.102 | SPII | HS |
| 152 | **Swarna** | 0.056 | 0.048 | 0.896 | SPIII | HS |

**Supplementary Table 5. Allele-frequency divergence among populations (net-nucleotide distance), computed using point estimates of P.**

|  | Sub-population 1 | Sub-population 2 | Sub-population 3 |
| --- | --- | --- | --- |
| Sub-population 1 | - | 0.050 | 0.069 |
| Sub-population 2 | 0.050 | - | 0.032 |
| Sub-population 3 | 0.069 | 0.032 | - |

**Supplementary Table 6. Average distances (expected heterozygosity) between individuals in same cluster**

| Sub-population 1 | 0.1077 |
| --- | --- |
| Sub-population 2 | 0.1217 |
| Sub-population 3 | 0.1023 |
| Mean alpha value (α) | 0.2010 |

**Supplementary Table 7. Pair-wise FST estimates among three populations of germplasm against *N. lugens***

|  | Resistant | Moderately resistant | Susceptible | Moderately susceptible | Highly susceptible |
| --- | --- | --- | --- | --- | --- |
| Resistant | 0.000 |  |  |  |  |
| Moderately resistant | 0.038 | 0.000 |  |  |  |
| Susceptible | 0.079 | 0.066 | 0.000 |  |  |
| Moderately susceptible | 0.102 | 0.090 | 0.079 | 0.000 |  |
| Highly susceptible | 0.097 | 0.074 | 0.072 | 0.110 | 0.000 |

**Supplementary Table 8. Candidate/functional genes associated with significant SSR markers from our study**

| **SN** | **Marker** | **Chromosome No** | **BPH Gene** | **Additional Functions** | **RAD ID*** |
| --- | --- | --- | --- | --- | --- |
| 1 | RM7 | 3 | *Qbph3* | Antibiosis mechanism | NA |
| 2 | RM1313 | 2 | *Bph9* | Antibiosis mechanism | NA |
